# A rationale for continuing mass antibiotic distributions for trachoma

**DOI:** 10.1186/1471-2334-7-91

**Published:** 2007-08-07

**Authors:** Kathryn J Ray, Travis C Porco, Kevin C Hong, David C Lee, Wondu Alemayehu, Muluken Melese, Takele Lakew, Elizabeth Yi, Jenafir House, Jaya D Chidambaram, John P Whitcher, Bruce D Gaynor, Thomas M Lietman

**Affiliations:** 1 Francis I. Proctor Foundation, University of California, San Francisco, USA; 2 Mathematics Department, San Francisco State University, USA; 3 Department of Public Health, San Francisco, USA; 4 Orbis International, Addis Ababa, Ethiopia; 5 Department of Ophthalmology, University of California, San Francisco, USA; 6 Department of Epidemiology & Biostatistics, University of California, San Francisco, USA; 7 Institute for Global Health, University of California, San Francisco, USA

## Abstract

**Background:**

The World Health Organization recommends periodic mass antibiotic distributions to reduce the ocular strains of chlamydia that cause trachoma, the world's leading cause of infectious blindness. Their stated goal is to control infection, not to completely eliminate it. A single mass distribution can dramatically reduce the prevalence of infection. However, if infection is not eliminated in every individual in the community, it may gradually return back into the community, so often repeated treatments are necessary. Since public health groups are reluctant to distribute antibiotics indefinitely, we are still in need of a proven long-term rationale. Here we use mathematical models to demonstrate that repeated antibiotic distributions can eliminate infection in a reasonable time period.

**Methods:**

We fit parameters of a stochastic epidemiological transmission model to data collected before and 6 months after a mass antibiotic distribution in a region of Ethiopia that is one of the most severely affected areas in the world. We validate the model by comparing our predicted results to Ethiopian data which was collected biannually for two years past the initial mass antibiotic distribution. We use the model to simulate the effect of different treatment programs in terms of local elimination of infection.

**Results:**

Simulations show that the average prevalence of infection across all villages progressively decreases after each treatment, as long as the frequency and coverage of antibiotics are high enough. Infection can be eliminated in more villages with each round of treatment. However, in the communities where infection is not eliminated, it returns to the same average level, forming the same stationary distribution. This phenomenon is also seen in subsequent epidemiological data from Ethiopia. Simulations suggest that a biannual treatment plan implemented for 5 years will lead to elimination in 95% of all villages.

**Conclusion:**

Local elimination from a community is theoretically possible, even in the most severely infected communities. However, elimination from larger areas may require repeated biannual treatments and prevention of re-introduction from outside to treated areas.

## Background

Trachoma remains the major cause of infectious blindness in the world [1,2]. Repeated infection with the ocular strains of *Chlamydia trachomatis *can lead to a cascade of conjunctival scarring, in-turned eyelids and eyelashes, and eventually blindness due to corneal opacity. To reduce the prevalence of infection, the World Health Organization (WHO) has recommended community-wide distributions of oral azithromycin as part of its strategy to control blinding trachoma by the year 2020[3]. They believe that infection can be reduced to a level low enough that resulting blindness will no longer be a major public health concern, but they do not believe that infection can be completely eliminated from an area.

A single dose of oral azithromycin is clearly effective in eliminating ocular chlamydial infection in an individual [4,5]. Antibiotic distributed simultaneously to an entire community has been shown to reduce the prevalence of infection to low levels [6-11]. The WHO recommends mass treatments be administered with an observed clinical prevalence greater than 10 percent[3]. Unfortunately, infection returns back into communities over time [7,12-15]. Repeated treatments have progressively reduced infection, at least in two areas with moderate disease. After three annual treatments, only a single infection could be identified in a village in western Nepal[8]. Similarly, after a single mass oral azithromycin distribution and three biannual topical tetracycline distributions, only a single infection was found in a Tanzanian village [9]. However, infection has never been eliminated from a hyper-endemic area, and if antibiotics are discontinued infection will presumably return. We have no proven long-term rationale for repeated mass antibiotic distributions in severely affected areas. Randomised controlled trials are the gold standard for assessing the effect of different treatment strategies, and several are currently underway. However, it is difficult with trials to explore the long term effects several years in the future for a range of strategies. Here, here we use short term data from Ethiopia to mathematically model longer term outcomes.

**Figure 1:**
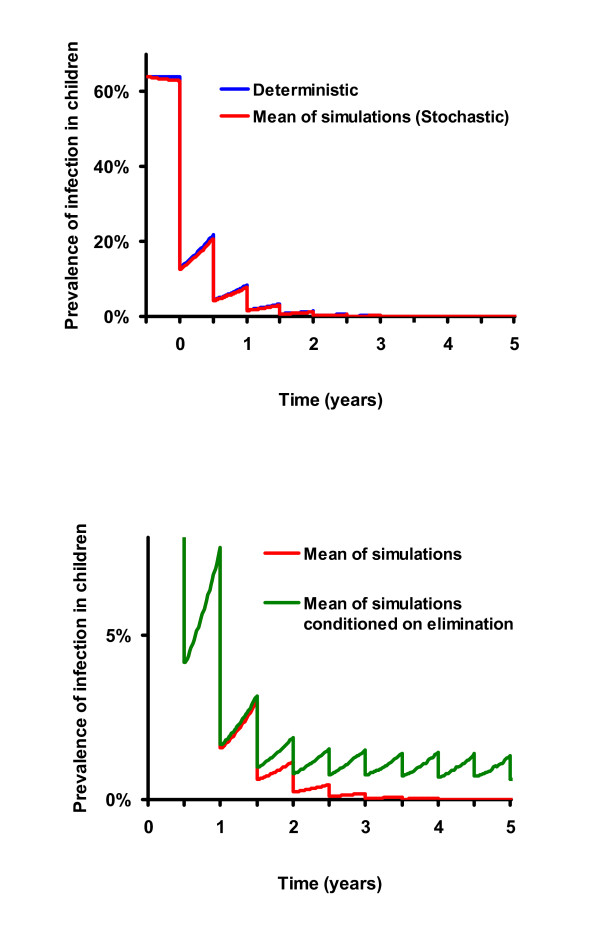
a. Deterministic Model of Time vs. Prevalence with biannual treatments: Results from a differential equation based model demonstrating that biannual coverage of 80% of the population should progressively reduce ocular chlamydial infection (blue curve). The deterministic model is an excellent approximation for the expectation of the stochastic model (mean of 1000 simulations, red curve). b. Stochastic Model of Time vs. Prevalence with biannual treatments: The mean of 1000 simulations of a stochastic model, assuming biannual treatments with 80% coverage (again, red curve) vs. the average prevalence of only those villages which still harbor infection (green curve). After the third treatment, the average prevalence of infection in these villages returns to approximately the same level with each subsequent treatment (green curve).

In previous studies, we have used deterministic models of difference [16] and differential equations [7] to model infection in communities. Mass antibiotic treatment is incorporated into the model by lowering infection by an amount proportional to the coverage of the community and the efficacy of antibiotic in an individual [7,16]. In the model, infection returns into a community between treatments according to logistic growth at a rate estimated from baseline data[16] or determined from post-treatment Ethiopian data [7]. The models suggest that periodic mass distributions can progressively reduce chlamydial infection in a community, as long as infection is reduced more by each treatment than it returns between treatments (Figure 1a) [7,16]. In this report, we use data collected from 16 villages in Ethiopia to estimate parameters for a mathematical model that incorporates the effects of chance. We then determine whether local elimination is possible, and if so, in what time span.

## Methods

### Clinical data

Sixteen villages in the Gurage region of southern Ethiopia were enrolled in a mass antibiotic treatment program for trachoma as previously described [7]. These villages are very remote with approximately 2 kilometres or more between them, thus we consider them to be closed communities. Briefly, 1–5 year old children, those most likely to harbor infection, were monitored at baseline and 2, 6, 12, 18, and 24 months after baseline. Every member of all sixteen villages>= 1 year old in the study were offered antibiotic treatments 1 week after baseline, 6,12, and 18 month visits. Pregnant women and those allergic to macrolides were offered topical tetracycline. The right upper conjunctiva of each child was swabbed and tested for the presence of chlamydial DNA using Amplicor PCR (Roche Diagnostics, Branchburg, NJ). Since post-treatment prevalence was expected to be relatively low, swabs from the same village were randomly pooled into groups of 5 with any remainder pooled into a final tube, as previously described [7,17]. The number of positive individual samples in a village most likely to have resulted in the observed pooled results was determined by maximum likelihood estimation.

### Models

Previously, we constructed a simple SIS (susceptible, infected, susceptible) model of ocular chlamydial infection in a core group of children using differential equations [18,19].

d(I)d(t)=β(N−I)IN−γI
 MathType@MTEF@5@5@+=feaafiart1ev1aaatCvAUfKttLearuWrP9MDH5MBPbIqV92AaeXatLxBI9gBaebbnrfifHhDYfgasaacH8akY=wiFfYdH8Gipec8Eeeu0xXdbba9frFj0=OqFfea0dXdd9vqai=hGuQ8kuc9pgc9s8qqaq=dirpe0xb9q8qiLsFr0=vr0=vr0dc8meaabaqaciaacaGaaeqabaqabeGadaaakeaadaWcaaqaaiabdsgaKjabcIcaOiabdMeajjabcMcaPaqaaiabdsgaKjabcIcaOiabdsha0jabcMcaPaaacqGH9aqpiiGacqWFYoGycqGGOaakcqWGobGtcqGHsislcqWGjbqscqGGPaqkdaWcaaqaaiabdMeajbqaaiabd6eaobaacqGHsislcqWFZoWzcqWGjbqsaaa@42D5@

where *I *is number of infectious cases, *t *is time, *γ *is the rate of recovery from infection, *β *is a transmission parameter, and *N *is the effective population size. Treatment was simulated by reducing the number of individuals infected by the product of the coverage, *c*, and the efficacy, *f*, of antibiotic in an individual (Figure 1a). For this study, a stochastic model (continuous time, discrete individuals) analogous to equation 1 having the transitions shown in Table 1 and using the parameters shown in Table 2 and was used.

**Table 1:** Transitions used in stochastic model

**Transition**	**Description of transition**	**Hazard/Risk**
S→I	susceptible becomes infected	*β*
I→S	infected recovers naturally	*γ*
I→S	periodic antibiotic treatment	c*f

**Table 2:** Parameters used in stochastic model

**Parameter**	**Definition**	**Value**	**Units**	**Estimated or Known**
N	Number of children in a village	100	people	Known
I	Number of children infected	varies by time	people	Known
*period*	Weeks between a mass treatment	26	weeks	Known
c*f	Effective Coverage	90%	percentage	Known
*γ*	Rate of recovery	0.017	1/weeks	Estimated using MLE
*β*	Rate of infection transmission	0.047	1/weeks	Estimated using MLE

Note that treatment is not given at a constant rate, but is given periodically to a certain proportion of the population. Each member of the community has a chance of being treated based on the coverage level and the efficacy of antibiotic in an individual.

We constructed the analogous Markov model by letting *p*_*i*_(*t*) denote the probability that there are *i *infected individuals in the population at time *t *(where *i *varies from 0 to *N*). Assume that the mass treatments happen at time 0,*T*,2*T*,...,*kT*,...; between periodic mass treatments, the model is a standard continuous time Markov process with constant coefficients. Assuming that infected individuals recover naturally at rate *γ*, and uninfected individuals become infected at rate *βI*/*N *leads us to the Kolmogorov forward equations, which are

dpidt=β(i−1)(N−i+1)N(1−δ0i)pi−1−(βi(N−i)N+iγ)pi+(i+1)γ(1−δNi)pi+1
 MathType@MTEF@5@5@+=feaafiart1ev1aaatCvAUfKttLearuWrP9MDH5MBPbIqV92AaeXatLxBI9gBaebbnrfifHhDYfgasaacH8akY=wiFfYdH8Gipec8Eeeu0xXdbba9frFj0=OqFfea0dXdd9vqai=hGuQ8kuc9pgc9s8qqaq=dirpe0xb9q8qiLsFr0=vr0=vr0dc8meaabaqaciaacaGaaeqabaqabeGadaaakeaadaWcaaqaaiabdsgaKjabdchaWnaaBaaaleaacqWGPbqAaeqaaaGcbaGaemizaqMaemiDaqhaaiabg2da9maalaaabaacciGae8NSdi2aaeWaaeaacqWGPbqAcqGHsislcqaIXaqmaiaawIcacaGLPaaadaqadaqaaiabd6eaojabgkHiTiabdMgaPjabgUcaRiabigdaXaGaayjkaiaawMcaaaqaaiabd6eaobaadaqadaqaaiabigdaXiabgkHiTiab=r7aKnaaBaaaleaacqaIWaamcqWGPbqAaeqaaaGccaGLOaGaayzkaaGaemiCaa3aaSbaaSqaaiabdMgaPjabgkHiTiabigdaXaqabaGccqGHsisldaqadaqaamaalaaabaGae8NSdiMaemyAaK2aaeWaaeaacqWGobGtcqGHsislcqWGPbqAaiaawIcacaGLPaaaaeaacqWGobGtaaGaey4kaSIaemyAaKMae83SdCgacaGLOaGaayzkaaGaemiCaa3aaSbaaSqaaiabdMgaPbqabaGccqGHRaWkdaqadaqaaiabdMgaPjabgUcaRiabigdaXaGaayjkaiaawMcaaiabeo7aNnaabmaabaGaeGymaeJaeyOeI0Iae8hTdq2aaSbaaSqaaiabd6eaojabdMgaPbqabaaakiaawIcacaGLPaaacqWGWbaCdaWgaaWcbaGaemyAaKMaey4kaSIaeGymaedabeaaaaa@75BF@

between the *k*th mass treatment and the next (*δ*_*ij *_is the Kronecker delta, which equals 1 if *i *= *j *and zero otherwise). At the mass treatment times, however, we assume that each infected individual has a probability *c *of being treated (the coverage), times a probability *f *of being cured if treated (the efficacy).

Parameters for this stochastic model were fit to the 6 month Ethiopian data using maximum likelihood estimation. We started out simulations at the average prevalence, and ran for 24 months to allow the distribution of prevalence to approximate the pre-treatment distribution at the time point zero We approximated the joint probability distribution function of the 3 points (baseline, 2, and 6 month post-treatment prevalence) by fitting a standard kernel density estimator (a 3-dimensional product-Epanechnikov kernel), to the results of 1000 simulations. The bandwidth of the kernel in each dimension was set using standard techniques [20]. We used the estimated likelihood to determine the log likelihood of the observed data. The values of the parameters *β *and *γ *which maximized the estimated likelihood were determined iteratively and uncertainty was estimated by inverting the Hessian of the log-likelihood function. Sensitivity analyses were performed by varying a single parameter at a time, keeping other parameters set at *β *= 0.044 and *γ *= 0.017, N = 100, effective coverage of 90%, and treatment frequency biannual.

**Figure 6:**
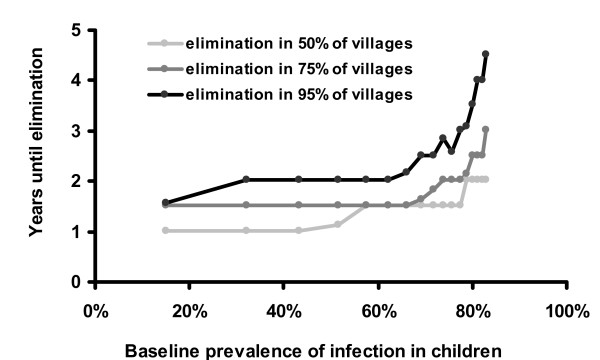
Baseline prevalence vs. Years until elimination: Here we vary *β *in the stochastic model, while keeping other parameters the same. Other parameters are biannual treatment with 90% effective coverage, an effective population size of 100 children, and *γ *= 0.017.

**Figure 7:**
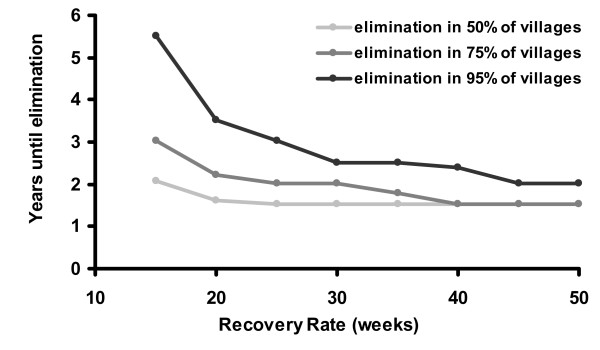
Recovery rate vs. Years until elimination: Here we vary the recovery rate in the stochastic model, while keeping other parameters the same. Other parameters are biannual treatment with 90% effective coverage, an effective population size of 100 children, and *β *= 0.044.

For the sensitivity analysis, we varied *γ *from 0.005 to 0.022, showing (Figure 7). We varied *β *from 0.01 to 0.1, illustrated in Figure 6, with the corresponding baseline prevalence estimated from the deterministic model (1−γβ
 MathType@MTEF@5@5@+=feaafiart1ev1aaatCvAUfKttLearuWrP9MDH5MBPbIqV92AaeXatLxBI9gBaebbnrfifHhDYfgasaacH8akY=wiFfYdH8Gipec8Eeeu0xXdbba9frFj0=OqFfea0dXdd9vqai=hGuQ8kuc9pgc9s8qqaq=dirpe0xb9q8qiLsFr0=vr0=vr0dc8meaabaqaciaacaGaaeqabaqabeGadaaakeaacqaIXaqmcqGHsisldaWcaaqaaGGaciab=n7aNbqaaiab=j7aIbaaaaa@31E3@). Density graphs from empirical data were smoothed for presentation using a narrow Gaussian kernel around each data point.

**Figure 3:**
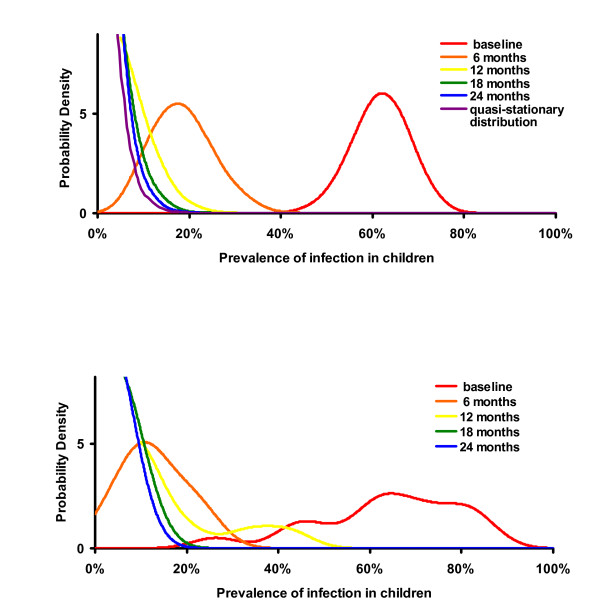
a. Probability Density of infection prevalence found in biannually treated villages where "village level" elimination has not yet occurred: Probability distribution of 1000 simulations at baseline, 6, 12, 18, and 24 months. The prevalence of infection in a simulated community pre-treatment varies in a normal distribution [21]. Each mass treatment eliminates infection in some villages, but in those that it does not, the distribution is shifted to the left, rapidly approaching a quasi-stationary distribution 3b. Probability density graphs using Ethiopian data: Distribution of the prevalence of infection in pre-school children in 16 Ethiopian villages. Baseline, 2, and 6 month data were used to fit the parameters of the stochastic model. Subsequent data from 12, 18, and 24 months confirm that the distribution of infection in the villages also approaches the quasi-stationary distribution.

To incorporate the effects of treatment, we used the analogous discrete time, Markov process where time is incremented every period of mass antibiotic distribution (e.g. 26 weeks). The quasi-stationary distribution (Figure 3a, violet curve) was determined by constructing a vector ***q***, with *q*_*i *_being the probability of having *i *infectious cases in the community (from i = 1 to the number of individuals in the community, *n*) and an *n *× *n *matrix *M *(determined from simulations), such that,

**M**·q^*t *^= q^*t*+treatment period^

Note that *q *is similar to *p *above, except that *q*_0, _the state that would represent elimination, is specifically not included in this formulation, so only communities where infection has not been eliminated are followed. The largest eigenvalue of *M *is the reciprocal of the expected time to extinction, and the corresponding eigenvector provides the quasi-stationary distribution (violet curve, Figure 3a) [21]. All models were built using Mathematica Version 5.1 using the statistical packages Discrete Distributions and Continuous Distributions.

## Results and Discussion

Parameters for a stochastic model were estimated from longitudinal data from approximately 5000 people within 16 separate Ethiopian villages during the first 6 months after a mass distribution. The MLE estimates for our model gave *β *= 0.044 and *γ *= 0.017. Gamma can be interpreted as the reciprocal of the duration of infection, and here would be approximately 1 year.

**Figure 2:**
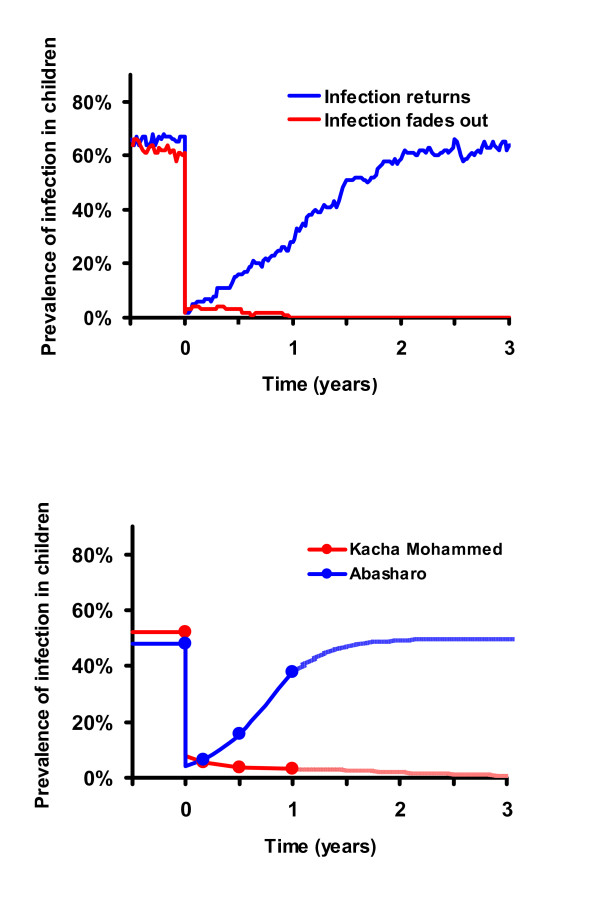
a. Simulation data after one treatment: In identical simulated communities, infection responds to identical treatment in different ways. It may return after a single mass antibiotic treatment relatively rapidly (blue curve) or fade out (red curve) due to the effects of chance. b. Real Ethiopian villages after one treatment: In Ethiopian communities with similar pre-treatment prevalence of infection and similar antibiotic coverage levels, infection may return relatively rapidly (blue curve), or fade out (red curve).

With estimated parameters, our mathematical models did indeed show that elimination is possible with repeated treatments. The stochastic element of the model recreates what we see happening in real life. In some villages we see infection returning, while in others it disappears. As more treatments are administered, we see more and more villages achieving elimination. Observing results from a 1000 simulations using the stochastic model, *the average *prevalence of infection is progressively reduced with each periodic treatment as in the deterministic model (Figure 1a and 1b, red curve). However, this reduction does not necessarily occur in all of the villages. In some simulated villages infection is eliminated with a single mass treatment. In others, it is reduced to a low level and fades out by chance (Figure 2a, red curve). In still others, infection may return quite rapidly (Figure 2b, blue curve). Simulations of 1000 communities demonstrate that infection is eliminated in more villages with each subsequent treatment. If not eliminated, it returns on average to the same level before the next scheduled treatment (green curve, Figure 1b). In fact, if we set aside the villages that have achieved elimination, the distribution of the prevalence of infection after the 3^rd ^treatment is similar to that after the 4^th ^treatment, and prevalence after the 4^th ^even more similar to that after the 5^th ^(Figure 3a). After two treatments, simulated communities quickly approach a distribution which, if conditioned on non-elimination, is stationary (termed a quasi-stationary distribution) [21-23]. This quasi-stationary distribution can be determined analytically (violet curve in Figure 3a, see methods).

Subsequent data from the same sixteen Ethiopian villages at 12, 18, and 24 months appear to confirm that elimination is possible. In one village, 50% of the children were infected at baseline, but no infection was identified in any of the 5 visits after the first treatment. In another, 45% of children were infected at baseline, but no infection could be found after the second treatment. If infection was not eliminated in a village then the distribution to which it returned 6 months after the 2^nd ^treatment approaches that to which it returns after the 3rd treatment (Figure 3b). There is no statistical difference between distributions 6 months after the 3^rd ^and 4^th ^treatments (Kolmogorov-Smirnov test, P = 0.95 comparing 18 and 24 month data).

This stochastic model does not incorporate re-introduction of infection from neighbouring villages. The rate that infection is re-introduced is not known and is difficult to estimate. Re-introduction appears not to be frequent, as communities do well in the short term, whether or not neighbouring villages were treated (unpublished data). On the other hand, it may well be occurring [13]. Some villages in which no infection could be found in children for several visits had infection identified at a subsequent visit. Even occasional transmission from other communities would prevent sustained elimination. Interestingly, the quasi-stationary distribution can be estimated from models by only considering villages where infection has occurred (the limiting case of the curves in Figure 3), by preventing the last infectious case from disappearing (violet curve in Figure 3a), or by artificially introducing a single infection into a community whenever infection has been eliminated, much as might be expected with re-introduction[21]. Since each of these methods mimics re-introduction, then the quasi-stationary distribution may be a reasonable estimate of the distribution of prevalence of infection.

Investigators are struggling to determine why infection returns in some villages and not others. This search is important, but may not be fruitful, since a great deal of variation is expected even in otherwise similar villages. Simulations allow us to construct communities that are absolutely identical, and variation is still observed due to the vagaries of who infects whom. Infection will be eliminated in some fortunate villages. In other, essentially identical villages, it will continue to return to the same average level even after multiple treatments (Figure 2a). In practice, we have also observed a varied response to treatment in similar villages (Figure 2b) [12].

Often overall prevalence is reported, but we believe there needs to be a shift in the way we evaluate progress. The average prevalence of infection across several villages can be misleading. A regional prevalence of 5% does not mean that 1 in 20 children in each village are infected. More likely, infection has been eliminated in most villages but has returned in a few villages to a level that may far exceed 5%. A better measure of success of a trachoma program may be the proportion of villages in which infection has been eliminated. While the power of sampling allows the mean prevalence to be obtained relatively easily, it should be interpreted in terms of what it reveals about the underlying process; after several rounds of treatment, the mean represents some villages where infection has been completely eliminated and others where infection has returned to a level chosen from the quasi-stationary distribution. Thus the decision for stopping distributions may need to depend on whether infection has been eliminated locally.

Periodic mass antibiotic treatments can locally eliminate the ocular chlamydia that cause blinding trachoma, if given frequently and to a large portion of the population [7,16]. Models suggest it will return to the same distribution before the next scheduled treatment. If infection is not eliminated from a community after several mass treatments, then stochastic Ethiopian data demonstrates that this quasi-stationary distribution is closely approached after only two or three treatments. The WHO's strategy requires only for antibiotics to reduce the level of infection and expects that other measures will be necessary for a permanent solution. Their current recommendation of three treatments before re-evaluation is reasonable. If constant re-infection of communities prevents elimination, then subsequent treatments may only serve to maintain the prevalence in a stationary distribution, not lower it. However, if infection between neighbouring communities is rare or can be reduced by treating large areas within a narrow time frame, then elimination over larger areas than a village may be achieved. There is some concern that communities from which trachoma has been partially eliminated will lose much of their immunity to chlamydia, only to have it return with a vengeance after treatments have been discontinued [24-26]. This has yet to be demonstrated convincingly in practice [26], but potential loss of immunity could easily be included in future models.

Partners in the WHO's trachoma program have distributed over 30 million doses of oral azithromycin so far, and over 1 million in Ethiopia. These programs are expanding rapidly. There has been evidence of subsequent re-emergence of infection in some recent trials of azithromycin, suggesting coverage and dosing intervals in mass therapy need careful consideration [7,14,24,27]. Biannual treatments are costly, require more resources, and may contribute to antibiotic resistance in chlamydia and other pathogens such as *Streptococcus pneumoniae *[26,28-30]. The benefits of mass therapy thus need to be weighed against the potential emergence of antibiotic resistance which will require long term surveillance. But if necessary for local elimination biannual distributions may be more cost effective in the long run in severely affected areas. The elimination of a bacterial disease from a large area with mass antibiotics would be major medical breakthrough.

Simulations were run to estimate the number of rounds of treatment necessary to achieve elimination in 50%, 75%, and 95% of the villages. Elimination depends on the efficacy and coverage of treatment, as well as the effective population size in a community. It should be noted that these models were developed using data from a hyper-endemic region, and they are not generalizable to all areas.

**Figure 4:**
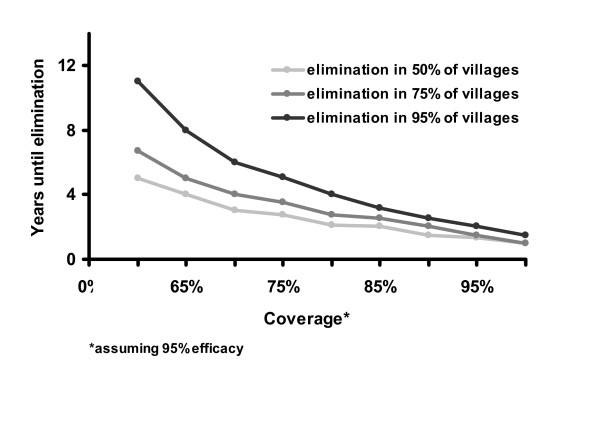
Coverage vs. Years until elimination: Here we vary coverage in the stochastic model, while keeping other parameters the same. Other parameters are biannual treatment an effective population size of 100 children, *γ *= 0.017, and *β *= 0.044.

**Figure 5:**
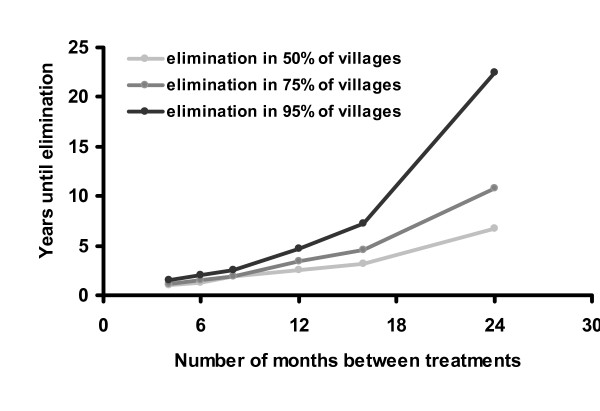
Number of months between treatments vs. Years until elimination: Here we vary treatment frequency in the stochastic model, while keeping other parameters the same. Other parameters are 90% effective coverage, an effective population size of 100 children, *γ *= 0.017, and *β *= 0.044.

**Figure 8:**
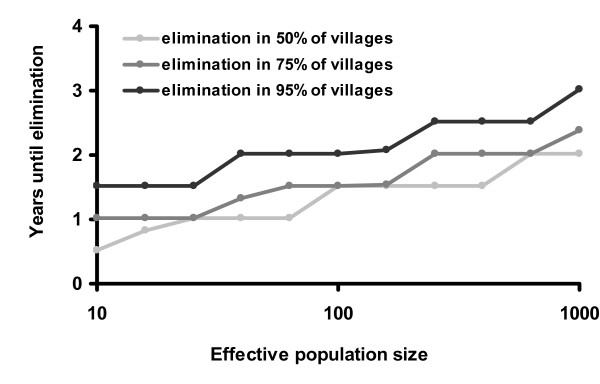
Population vs. Years until elimination: Here we vary the effective population size of children in the stochastic model, while keeping other parameters the same. Other parameters are biannual treatment with 90% effective coverage, *γ *= 0.017, and *β *= 0.044.

The stochastic model suggests that treatment given to 90% of the population biannually would eliminate infection in 95% of the villages after 5 years. Lower coverage or less frequent treatments would not be as successful (Figures 4 and 5). For example, the WHO's recommended strategy of annual treatment of 80% of the population would eliminate infection in an estimated 95% of hyper-endemic villages in 12 years. Not surprisingly, areas with a lower baseline prevalence of infection should achieve elimination more rapidly (Figure 6). If the rate that an individual recovers from an infection is more rapid than estimated here, then it will be more difficult to eliminate infection with repeated treatments (Figure 7). Infection can be eliminated from smaller communities more rapidly than larger ones, in part because stochastic fade out is more likely with fewer cases (Figure 8). Other factors, such as the cost of medication and of distributions, were not analyzed in this report, but clearly play a role in determining the optimal strategy for a given program.

## Conclusion

The local elimination of infectious trachoma is possible with repeated mass antibiotic distributions. A stochastic mathematical model suggests that elimination (reduction of the prevalence of infection to zero in a community) can occur within a reasonable time period even in the most severely affected areas, as long as the coverage and frequency of distributions are high enough. For example, in hyper-endemic areas of Ethiopia, we estimate that biannual treatment with 90% effective coverage would result in elimination in more than half of communities in 3 years, and in 95% within 5 years. Most areas have far less trachoma than the 16 villages in Ethiopia presented here, so elimination should prove far easier. The WHO's current recommendation of three annual treatments with at least 80% coverage will likely eliminate infection in a large number of communities worldwide. The time to elimination is also dependent on whether transmission of infection can be effectively reduced by any other means, such as hygiene (clean faces are associated with less clinically active trachoma) and fly control (Musca sorbens is thought to play a role in the transmission of ocular chlamydia), both of which are major parts of the WHO's overall trachoma strategy [31-34]. Researchers have noted that trachoma is disappearing in the absence of active trachoma programs [35-38]; such a secular trend would also make infection easier to eliminate.

## Competing interests

The author(s) declare that they have no competing interests.

## Authors' contributions

KR acquisition of data, mathematical modelling, analysis and interpretation of data, drafting of the manuscript, critical revision of the manuscript for important intellectual content, statistical analysis.

TP study concept and design, analysis and interpretation of data, drafting of the manuscript, critical revision of the manuscript for important intellectual content, statistical analysis.

KH acquisition of data, analysis and interpretation of data, critical revision of the manuscript for important intellectual content, administrative, technical, or material support, study supervision.

DL mathematical modeling, analysis and interpretation of data, critical revision of the manuscript for important intellectual content, statistical analysis.

WA study concept and design, acquisition of data, critical revision of the manuscript for important intellectual content, obtained funding.

MM study concept and design, acquisition of data, critical revision of the manuscript for important intellectual content, obtained funding.

TL study concept and design, acquisition of data, critical revision of the manuscript for important intellectual content, obtained funding.

EY acquisition of data, critical revision of the manuscript for important intellectual content, administrative, technical, or material support, study supervision.

JH acquisition of data, critical revision of the manuscript for important intellectual content, administrative, technical, or material support, study supervision.

JC acquisition of data, analysis and interpretation of data, critical revision of the manuscript for important intellectual content.

JW study concept and design, acquisition of data, drafting of the manuscript, critical revision of the manuscript for important intellectual content, obtained funding.

BG study concept and design, acquisition of data, critical revision of the manuscript for important intellectual content.

TL conceived of the study concept and design, acquisition of data, mathematical modelling, drafting of the manuscript, critical revision of the manuscript for important intellectual content, statistical analysis, obtained funding, study supervision.

## Pre-publication history

The pre-publication history for this paper can be accessed here:


